# Correlation between Bronchopulmonary Dysplasia and Cerebral Palsy in Children: A Comprehensive Analysis Using the National Inpatient Sample Dataset

**DOI:** 10.3390/children11091129

**Published:** 2024-09-18

**Authors:** Abdulrahman Al-Matary, Sameh Abozaid, Mustafa Al Suliman, Mohammed Alsubaie, Faisal K Aldandan, Faisal Mohammed Alzehairi, Huda Yahya Alyahyawi, Abrar Nayel Alsharief, Ghadeer Ghazi Alahmadi, Faris Althubaiti, Naseem Alyahyawi, Ahlam Mazi, Ahmed Abu-Zaid, Hind Alnajashi, Reem Abdullah Alyoubi

**Affiliations:** 1 Department of Neonatology, King Fahad Medical City, Riyadh 11525, Saudi Arabia; 2 Department of Neonatology, Maternity and Children Hospital, Madinah 42313, Saudi Arabia; 3 Department of Neonatology, Alyamamah Hospital, Riyadh 14222, Saudi Arabia; 4 Department of Medicine, King Abdulaziz University, Jeddah 11461, Saudi Arabia; 5 Department of Pediatrics, King Abdulaziz University, Jeddah 11461, Saudi Arabia; 6 Department of Biochemistry and Molecular Medicine, College of Medicine, Alfaisal University, Riyadh 11533, Saudi Arabia; 7 Department of Neurology, King Abdulaziz University, Jeddah 11461, Saudi Arabia

**Keywords:** cerebral palsy, bronchopulmonary dysplasia, pediatrics, National Inpatient Sample, prematurity

## Abstract

**Background**: The existing literature lacks conclusive evidence regarding the relationship between bronchopulmonary dysplasia (BPD) and cerebral palsy (CP). This large epidemiological study aimed to explore the co-occurrence of BPD and CP among children. **Methods**: This retrospective cohort analysis utilized the National Inpatient Sample (NIS) dataset from 2016 to 2019, investigating pediatric patients with BPD and CP diagnoses. Descriptive and inferential statistics, including univariate and multivariate regression analyses, were conducted to explore the association between BPD and CP. **Results**: Overall, 3,951,039 patients were analyzed. Among them, 28,880 patients had CP (n = 796 with BPD and n = 28,084 without BPD). The rates of intraventricular hemorrhage grade 3 and 4, central nervous system anomalies, chromosomal disorders, retinopathy of prematurity (≥grade 3), periventricular leukomalacia, prematurity, and low birth weight were significantly higher in the CP-with-BPD arm contrasted to the CP-without-BPD arm. Univariate regression demonstrated a significant BPD–CP association (odds ratio [OR] = 7.78, 95% confidence interval [CI]: 7.24–8.37, *p* < 0.0001). Multivariate analysis, adjusting for various confounders, reinforced this association (OR = 5.70, 95% CI: 5.17–6.28, *p* < 0.0001). We observed a significant association between increasing prematurity in neonates with BPD and an elevated risk of CP. **Conclusions**: This nationwide study identified a strong correlation between the co-occurrence of BPD and CP, though it does not establish causality. Rigorous adjustments revealed that patients with BPD appear to have a six-fold increased likelihood of being diagnosed with CP later on, compared to those without BPD. While aligned with the existing literature, this study represents the largest sample size with recommendations for targeted preventive strategies to mitigate the burden of CP.

## 1. Introduction

Cerebral palsy (CP) is a collection of illnesses marked by lasting motor dysfunction involving movement, posture, and muscle tone. The occurrence of CP is alarming, impacting an approximated 2–3 per 1000 live births annually, highlighting its significant public health concern [1]. CP is frequently diagnosed around two years of age or later, largely because about 30% of children with CP do not show significant abnormalities on brain magnetic resonance imaging (MRI) [2,3,4,5]. This delay in diagnosis is due to the challenge of differentiating between mild CP symptoms and normal developmental milestones [6]. The diagnosis of CP typically involves a series of evaluations, including magnetic resonance imaging, to rule out other disorders and confirm the presence of CP [1,6].

The etiology of CP lies in injuries or abnormalities during brain development, with recognized risk factors including prematurity, infections, chromosomal disorders, genetic anomalies, and hypoxia [7]. Researchers have shown the complex association between CP and various neonatal factors, including bronchopulmonary dysplasia (BPD), which has been identified as an independent risk factor for CP and various neurodevelopmental impairments in pediatric patients [8,9].

BPD, a chronic lung disease necessitating oxygen therapy, predominantly impacts premature newborns. It leads to lung and bronchi damage, causing dysplasia in the alveoli. The documented global incidence spans from 10% to 89%, with regional variations [10]. Despite advances in care improving survival rates, the incidence of BPD has plateaued or even risen [11], underscoring its systemic impact and the significant healthcare expenses linked to long-term complications [12].

Investigating the connection between CP and BPD proves intricate due to the shared risk factors that elevate the risk of both conditions. These factors include extremely premature birth, extended use of mechanical ventilation, and cerebral white matter insult [8,13]. While existing studies propose an increased risk of neonatal brain injury and succeeding occurrence of CP in infants with BPD [8,9], the corresponding literature lacks conclusive evidence. The previous meta-analysis by Gou and colleagues, which highlighted BPD–CP association, has certain limitations [8]. For instance, the meta-analysis was grounded on a small sum of included studies and an uncertain definition of BPD, and more than half of these studies did not adjust for potential confounders, making the pooling result of the meta-analysis inconclusive [8]. In this study, we fill the gap by conducting this population-based study with a larger sample size and better adjustments for the confounding utilization of the National Inpatient Sample (NIS) [14]. Our objective is to contribute to the literature by addressing potential inconsistencies in the results often associated with limited sample sizes in BPD studies and enhancing the generalizability of findings.

## 2. Methods

This retrospective cohort analysis utilized the NIS database from U.S. hospitals, covering the four years from 2016 to 2019 [14]. The NIS, a component of the Healthcare Cost and Utilization Project, is the largest publicly accessible, all-payer inpatient database supported by the Agency for Healthcare Research and Quality. It encompasses histories of individuals managed in U.S. community hospitals, covering over 97% of the U.S. people. Representing an approximately 20% stratified cohort of discharges from community hospitals, the NIS compiles thirty-five million weighted hospitalizations across the U.S. Diagnoses were coded using the International Classification of Diseases 10th Revision, Clinical Modification (ICD-10-CM), as depicted in Supplemental Table S1. This research was granted exemption from the Institutional Review Board, given that the NIS is a publicly accessible archive containing anonymous patient information. Patients with BPD (independent variable) and CP (dependent variable) diagnoses were identified using ICD-10-CM/PCM codes P27.1 and G8, respectively, with a focus on the pediatric population aged less than 18 years.

We extracted baseline characteristics data such as sex, race, median household income, primary payer, hospital location, hospital teaching status, and hospital region. The other variables of interest used in the multivariable regression comprised central nervous system anomalies, chromosomal disorders, periventricular leukomalacia, intraventricular hemorrhage (grades 3 and 4), retinopathy of prematurity (≥grade 3), birth weight, and prematurity (based on gestational age).

STATA x15 was used to conduct our analyses. Descriptive statistics were utilized to depict the numbers of baseline characteristics, along with percentages in CP-without-BPD patients versus CP-with-BPD patients. Inferential statistics were used to investigate possible causality as well as univariate and multivariate regression analyses. Findings were displayed in odds ratios (ORs), along with their corresponding 95% confidence intervals (CIs). Sex, central nervous system anomalies, chromosomal disorders, periventricular leukomalacia, intraventricular hemorrhage (grades 3 and 4), retinopathy of prematurity (≥grade 3), low birth weight (<2500 g), and prematurity (<37 gestational weeks) were considered in the multivariate regression analysis model to adjust the possible bias of coexisting conditions. The Chi-square test (x^2^) was utilized to assess the statistical significance of differences across the two groups (CP without versus with BPD) for categorical variables. Regarding continuous variables (i.e., age and length of hospital stay), data were evaluated for normality by the Shapiro–Wilk test. The Student’s t-test and Mann–Whitney U test were used if the data were normally and not normally distributed, respectively. For all purposes, results were considered significant if the *p*-value measured < 0.05.

## 3. Results

We excluded patients with missing data (n = 1,641,602 admissions) and those above 18 years old (n = 22,888,957 patients). Overall, 3,951,039 patients were analyzed from the 2016–2019 NIS datasets. Among them, 3,922,159 patients had no CP (n = 14,224 with BPD and n = 3,907,935 without BPD). The baseline characteristics of all pediatric patients with CP (n = 28,880) based on the coexisting presence (n = 796) or absence (n = 28,084) of BPD are displayed in Table 1. The rates of intraventricular hemorrhage grades 3 and 4, central nervous system anomalies, chromosomal disorders, retinopathy of prematurity (≥grade 3), periventricular leukomalacia, prematurity, and low birth weight were significantly higher in the CP-with-BPD arm contrasted to the CP-without-BPD arm. Also, the length of hospital stay was significantly higher in the CP-with-BPD arm contrasted to the CP-without-BPD arm (10.06 ± 27.90 vs. 6.41 ± 11.36 days, *p* < 0.0001).

The univariate regression analysis reveals a substantial association between CP and BPD (OR = 7.78, 95% CI: [7.24, 8.37], *p* < 0.0001). This indicates that patients with BPD have 7.78-fold odds of having a diagnosis of CP compared to those without BPD. The *p*-value is highly significant, providing valuable insights into the unadjusted relationship between these two conditions within our study cohort. The multivariate regression analysis reveals several significant associations, as shown in Table 2. Patients with BPD were found to have a substantially higher likelihood of being diagnosed with CP (OR = 5.70, 95% CI: [5.17, 6.28], *p* < 0.0001). Female gender is associated with a decreased probability of having a diagnosis of CP (OR = 0.74, 95% CI: [0.72, 0.76], *p* < 0.0001). Additionally, the presence of central nervous system anomalies, periventricular leukomalacia, chromosomal disorders, and prematurity are all significantly associated with an increased probability of having a co-occurrence of CP. Conversely, conditions such as retinopathy of prematurity (grade ≥ 3), intraventricular hemorrhage (grades 2 and 3), and low birth weight are associated with lower odds of receiving a diagnosis of CP. Furthermore, the degree of prematurity is also a significant factor, with a higher probability of CP associated with more extreme prematurity. These findings highlight the complex interplay of various medical conditions and perinatal factors in the development of CP.

## 4. Discussion

The association between BPD and CP has been a topic of interest in several studies. However, the available research lacks a comprehensive exploration of the link between BPD and CP, necessitating further investigation to provide a clearer understanding. The prior meta-analysis conducted by Gou et al. depicts a significant correlation between CP and BPD (OR = 2.10, 95% CI: 1.57, 2.82) [8]. However, this meta-analysis has several shortcomings that ought to be acknowledged: notably, it relies on a small number of included studies with small sample sizes, lacks a clear definition of BPD, and over half of these studies do not account for potential confounders, thereby rendering the pooled results indecisive. Based on this, we hypothesized a potentially stronger association, so we performed this nationwide study to apply the analysis to a bigger sample size, with wider population characteristics and with better control for confounders. In our study, the logistic regression of the univariate analysis was performed on nearly four million admissions, and shows that patients with BPD have a significant eight-fold risk of developing CP compared to those without BPD. Even after adjusting for a large panel of key confounders, this association persisted, reinforcing our conclusion (patients with BPD have a significant six-fold risk of developing CP compared to those without BPD). From the multivariate regression findings, we also observed a progressive increase in the risk of CP with greater prematurity in neonates with BPD. This observation is in line with a secondary analysis published by Smith et al., which documents that the prevalence of CP was 13% at 24–26 gestational weeks, 8% at 26–28 gestational weeks, 4% at 28–30 gestational weeks, and roughly 1% afterward. Several lines of research highlight that the risk of developing CP increases with each added week of prematurity among infants with BPD [15,16,17].

In our study, the rate of 796 BPD cases per 28,800 CP cases (~3%) seems low. The incidence of BPD among patients with CP varies based on several factors, including the severity of CP and the presence of other medical conditions such as prematurity. Although BPD is not a direct complication of CP, both conditions are often associated with premature birth. Evidence indicates that premature infants are at a higher risk for both BPD and CP compared to full-term infants [8,16], which is consistent with the data presented in Table 1. Studies show that among children with CP, especially those born prematurely, the prevalence of BPD can be significant. For instance, in very preterm infants who later develop CP, the incidence of BPD can range from 20% to 50% [16]. The relatively low prevalence of BPD in our CP patients may be attributed to the lower rate of prematurity, including fewer cases of early labor delivery and low birth weight, among these patients.

In the current investigation, the occurrence of CP in the study population (28,800 per 3,951,039, ~7 per 1000) was higher than the published incidence data for CP, which are generally in the range of 1–4 per 1000. It should be noted that the NIS is a large administrative database that includes data on hospitalized patients, and it may reflect a higher prevalence of CP due to its focus on those requiring inpatient care. Patients in this database are often those with more severe or complex cases of CP, as they are more likely to be hospitalized, compared to individuals with milder forms of the condition, who do not require such care. Thus, inclusion of those high-risk CP patients for hospitalization could skew the data and lead to a higher observed prevalence of CP compared to the incidence and prevalence rates reported in broader population-based studies. This hospitalization bias underscores the need for careful interpretation of the data, acknowledging that the NIS may not fully represent the general population’s experience with CP.

Numerous investigations have suggested that lung–brain interaction injuries may encompass intricate mechanisms including inflammation, oxidative stress, neural degeneration, angiogenesis, and architectural remodeling [18,19,20]. Additionally, subsequent inflammatory activation of oligodendrocytes can culminate in disruptions in myelination, followed by demyelination and white matter injury [21,22]. Also, intermediations for BPD management, like mechanical ventilation and postnatal corticosteroids, have been linked to subsequent brain damage [16,23,24,25]. These pathways highlight the multifactorial nature of how BPD affects neurodevelopmental outcomes and increases the risk of CP in affected infants.

Based on our findings, it is imperative for clinicians to promptly identify and address BPD in infants to mitigate the likelihood of CP development. Failure to promptly diagnose CP in high-risk infants could lead to missed opportunities for interventions during a critical period of neuroplasticity. Treatment approaches targeting BPD during both the antenatal and postnatal periods have been shown to decrease the likelihood of CP [7]. Although multiple courses of antenatal steroids have demonstrated a reduction in both CP and preterm delivery risks, it is vital to recognize that a single regimen is generally considered the preferred standard of care [26]. Other postnatal therapies include surfactant replacement therapy, vitamin A, oxygen therapy, and caffeine, which can reduce the necessity for mechanical ventilation in preterm infants with BPD [27]. A multicenter study has recommended managing BPD with supplemental oxygen without mechanical ventilation, as it is not linked to an augmented hazard of any CP phenotype, contrasted with oxygen with mechanical ventilation, which elevates the risk of CP nearly sixfold [16].

There may be a temporal relationship where a diagnosis of BPD often precedes a diagnosis of CP, as CP can be challenging to diagnose early in life. The presence of BPD may increase the likelihood of a CP diagnosis later. Therefore, the observed association between BPD and CP may reflect a co-occurrence of these conditions, particularly among infants born prematurely or those with more severe CP. It is possible that BPD might exacerbate the severity of CP without directly causing it. For instance, premature infants who experience hemorrhagic events may develop CP, BPD, or both, and among patients with BPD and CP, the presence of BPD could potentially worsen the severity of CP.

Our study provides correlations between BPD and CP but does not establish causality. To establish that a specific factor, such as BPD, is a true risk factor for developing CP, a controlled prospective study is necessary. In such a study, BPD should be measured before the onset of CP to accurately establish a temporal relationship between the risk factor and the disease. By tracking individuals over time and comparing those with and without BPD, researchers can assess whether BPD increases the likelihood of developing CP, thereby providing robust evidence of causality. This approach ensures that the risk factor is assessed prior to the disease development, which is crucial for confirming its role as a true risk factor.

This study gives valuable insights into the complex relationship between BPD and CP, utilizing a large and diverse NIS dataset from 2016 to 2019. The nationwide representation of the data, comprising over 15,020 patients diagnosed with BPD, provides a solid foundation for analysis, enhancing the generalizability of the findings. The comprehensive nature of this study is evidenced by the meticulous inclusion of detailed demographic, socioeconomic, and clinical variables, allowing for a nuanced exploration of potential associations and confounding factors. The statistical rigor applied, with both univariate and multivariate regression analyses, strengthens this study’s methodology, revealing a substantial association between BPD and CP. Even after adjusting for potential confounders in the multivariate regression, this association persists, emphasizing the robustness of the observed relationship.

The current study has several intrinsic limitations that should be acknowledged. Such limitations include the retrospective nature of this study, which constrains our ability to establish causality and determine the definitive temporality between BPD and CP. Furthermore, the lack of detailed clinical information in the NIS, such as motor function classification of CP and long-term follow-up data, restricts our ability to fully assess the outcomes and progression of BPD and CP over time. Additionally, we did not assess the correlation between different types of CP and BPD, which could have provided more precise correlative data. Additionally, the definition of the ICD-10 code P27.1 simply indicates “BPD originating in the perinatal period” without providing specific details. For example, it does not specify whether BPD is defined by oxygen dependency at 36 weeks postmenstrual age, a duration of 28 days of oxygen dependency during hospitalization, or severe BPD requiring mechanical ventilation at 36 weeks, which could affect the interpretation of results and their correlations.

Despite its considerable utility in large-scale epidemiological research, the NIS comes with inherent limitations that warrant careful consideration. The exclusive focus on inpatient data poses challenges in generalizing findings to outpatient settings, potentially introducing bias towards more severe cases. Coding accuracy and variability across healthcare institutions may impact the precision of reported data, while the lack of detailed clinical information restricts the depth of analysis. Sampling bias and the inability to confirm the accuracy of diagnoses are additional concerns. Moreover, the NIS’s specific focus on the U.S. population raises questions about the international applicability of findings.

## 5. Conclusions

This nationwide study identifies a strong correlation between the co-occurrence of BPD and CP, though it does not establish causality. Rigorous adjustments reveal that patients with BPD appear to have a six-fold increased likelihood of being diagnosed with CP later, compared to those without BPD. These findings, derived from the comprehensive NIS dataset, yield nuanced insights, paving the way for targeted interventions and enhancing our understanding of the complex relationship between BPD and CP for improved patient outcomes.

## Figures and Tables

**Table 1:** Prevalence and distribution of the demographics of patients hospitalized with a diagnosis of cerebral palsy with and without bronchopulmonary dysplasia.

Variables	CP without BPD (n = 28,084)	CP with BPD (n = 796)	*p*-Value
Age in years (mean ± SD)	9.52 ± 5.11	5.34 ± 4.37	<0.0001
Length of hospital stay in days (mean ± SD)	6.41 ± 11.36	10.06 ± 27.90	<0.0001
Death	263 (0.94%)	7 (0.88%)	0.869
Sex			0.076
Male	16,156 (57.53%)	483 (60.68%)	
Female	11,928 (42.47%)	313 (39.32%)	
Primary expected payer			0.243
Medicare	90 (0.32%)	3 (0.38%)	
Medicaid	18,043 (64.25%)	522 (65.58%)	
Private insurance	8020 (28.56%)	234 (29.40%)	
Self-Pay	230 (0.82%)	6 (0.75%)	
No charge	13 (0.05%)	0 (0.00%)	
Other	1688 (6.01%)	31 (3.89%)	
Race			<0.0001
White	13,503 (48.08%)	334 (41.96%)	
Black	5326 (18.96%)	266 (33.42%)	
Hispanic	6652 (23.69%)	149 (18.72%)	
Asian or Pacific Islander	986 (3.51%)	20 (2.51%)	
Native American	230 (0.82%)	5 (0.63%)	
Other	1387 (4.94%)	22 (2.76%)	
Calendar year			
2016	6379 (22.71%)	138 (17.34%)	
2017	7051 (25.11%)	207 (26.01%)	
2018	7296 (25.98%)	227 (28.52%)	
2019	7358 (26.20%)	224 (28.14%)	
Median household income quartile			0.556
1st-25th	8383 (29.85%)	253 (31.78%)	
26th-50th	7585 (27.01%)	201 (25.25%)	
51st-75th	6754 (24.05%)	195 (24.90%)	
76th-100th	5362 (19.09%)	147 (18.47%)	
Bed size of hospital			0.001
Small	5715 (20.35%)	202 (25.38%)	
Medium	5590 (19.90%)	163 (20.48%)	
Large	16,779 (59.75%)	431 (54.15%)	
Location/teaching status of hospital			0.133
Rural	339 (1.21%)	6 (0.75%)	
Urban non-teaching	1049 (3.74%)	21 (2.64%)	
Urban teaching	26,696 (95.06%)	769 (96.61%)	
Region of Hospital			<0.0001
Northeast	4292 (15.28%)	121 (15.20%)	
Midwest or North Central	6777 (24.13%)	257 (32.29%)	
South	10,185 (36.27%)	284 (35.68%)	
West	6830 (24.32%)	134 (16.83%)	
Intraventricular Hemorrhage (grades 3, 4)			<0.0001
No	28,071 (99.95%)	785 (98.62%)	
Yes	13 (0.05%)	11 (1.38%)	
Central Nervous System Anomalies			0.006
No	21,887 (77.93%)	653 (82.04%)	
Yes	6197 (22.07%)	143 (17.96%)	
Chromosomal disorders			0.011
No	26,634 (94.84%)	771 (96.86%)	
Yes	1450 (5.16%)	25 (3.14%)	
Periventricular leukomalacia			<0.0001
No	27,939 (99.48%)	774 (97.24%)	
Yes	145 (0.52%)	22 (2.76%)	
Retinopathy of prematurity (grade ≥3)			<0.0001
No	28,076 (99.97%)	787 (98.87%)	
Yes	8 (0.03%)	9 (1.13%)	
Patent ductus arteriosus			<0.0001
No	27,946 (99.51%)	769 (96.60%)	
Yes	138 (0.49%)	27 (3.40%)	
Prematurity			<0.0001
Full term (≥37 gestational weeks)	27,479 (97.84%)	647 (81.28%)	
Moderate to late (32 to <37 gestational weeks)	135 (0.48%)	8 (0.63%)	
Very preterm (28 to <32 gestational weeks)	160 (0.58%)	11 (1.13%)	
Extremely preterm (<28 gestational weeks)	288 (1.03%)	127 (15.95%)	
Unspecified gestational age	22 (0.07%)	3 (0.63%)	
Birth Weight			<0.0001
Normal or high birth weight (≥2.5 kg)	28,040 (99.84%)	775 (97.36%)	
Moderately low birth weight (2.5–1.5 kg)	13 (0.05%)	2 (0.25%)	
Very low birth weight (1.5–1 kg)	11 (0.04%)	3 (0.38%)	
Extremely low birth weight (<1 kg)	20 (0.07%)	16 (2.01%)	

**Table 2:** Multivariate association between various demographic and clinical perinatal variables and the development of cerebral palsy.

Variable	OR	Lower CI	Upper CI	*p*-Value
Bronchopulmonary dysplasia	5.70	5.17	6.28	<0.0001
Female gender	0.74	0.72	0.76	<0.0001
Intraventricular hemorrhage (grades 3 and 4)	0.10	0.06	0.16	<0.0001
Central nervous system anomalies	29.79	28.87	30.74	<0.0001
Chromosomal disorders	2.59	2.43	2.75	<0.0001
Periventricular leukomalacia	10.30	8.25	12.87	<0.0001
Retinopathy of prematurity (grade 3 or more)	0.19	0.11	0.35	<0.0001
Moderate low birth weight	0.03	0.02	0.05	<0.0001
Very low birth weight	0.01	0.008	0.02	<0.0001
Extremely low birth weight	0.01	0.01	0.02	<0.0001
Moderate-to-late preterm	0.13	0.11	0.15	<0.0001
Very preterm	1.83	1.53	2.19	<0.015
Extremely preterm	3.77	3.29	4.32	<0.0001

OR: odds ratio; CI: confidence interval.

## Data Availability

Data were obtained from a publicly available database under the Hospital Cost and Utilization Project’s Nationwide Inpatient Sample, which can be accessed from the following: https://www.hcup-us.ahrq.gov/db/nation/nis/nisdbdocumentation.jsp (accessed on 18 July 2024).

## Supplementary Materials

The following supporting information can be downloaded at: https://www.mdpi.com/article/10.3390/children11091129/s1, Table S1: The ICD-10 codes and NIS demographics were used in the analysis.
