# Supplementary material for: Correlation between Bronchopulmonary Dysplasia and Cerebral Palsy in Children: A Comprehensive Analysis Using the National Inpatient Sample Dataset
Source: Children (Basel). 2024 Sep 18;11(9):1129. doi: 10.3390/children11091129 (PMC11430353; doi:10.3390/children11091129)
Supplement: Supplementary file 1 [file children-11-01129-s001.zip › children-3188010-supplementary.pdf]

Supplemental Table 1. The ICD-10 codes and NIS demographics were used in the analysis.

**Variable**	**Source**	**ICD-10-CM/PCD code**
Bronchopulmonary dysplasia	I10_DX1/40	P271
Cerebral palsy	I10_DX1/40	G80
Intraventricular hemorrhage grade 3 and 4	I10_DX1/40	P522
Congenital anomalies of the central nervous system	I10_DX1/40	Q0
Chromosomal disorders	I10_DX1/40	Q9
Periventricular leukomalacia	I10_DX1/40	P912
Retinopathy of prematurity grade 3 or higher	I10_DX1/40	H3514, H3515, H3516
Patent ductus arteriosus	I10_DX1/40	Q25.0
Extremely preterm (less than 28 weeks)	I10_DX1/40	P07.2
Very preterm (28 to 31 weeks)	I10_DX1/40	P0731, P0732, P0733, P0734
Moderate to late preterm (32 to 36 weeks)	I10_DX1/40	P0735, P0736, P0737, P0738, P0739
Unspecified gestational age	I10_DX1/40	P0730
Extremely low birth weight (<1000 g)	I10_DX1/40	P070
Very low birth weight (1000–1499 g)	I10_DX1/40	P0714, P0715
Moderate low birth weight (1500–2499 g)	I10_DX1/40	P0716, P0717, P0718
Age	NIS Core/Hospital	-
FEMALE	NIS Core/Hospital	-
Primary expected payer	NIS Core/Hospital	-
Race	NIS Core/Hospital	-
Year	NIS Core/Hospital	-
ZIP income quartile	NIS Core/Hospital	-
Location/teaching hospital status	NIS Core/Hospital	-
Hospital bed size	NIS Core/Hospital	-
Length of hospital stay	NIS Core/Hospital	-
Death	NIS Core/Hospital	-

NIS: National Inpatient Sample; ICD-10-CM/PCD: the 10th revision of the International Classification of Diseases, Clinical Modification/Procedure Coding System
